# Communication is the key: biofilms, quorum sensing, formation and prevention

**DOI:** 10.15190/d.2019.13

**Published:** 2019-09-30

**Authors:** Veronica G. Preda, Oana Săndulescu

**Affiliations:** Department of Biochemistry and Molecular Biology, University of Bucharest, Faculty of Biology, Bucharest, Romania; Department of Genetics and Applied Biotechnology, University of Bucharest, Faculty of Biology, Bucharest, Romania; Department of Infectious Diseases I, Carol Davila University of Medicine and Pharmacy, National Institute for Infectious Diseases “Prof. Dr. Matei Balș”, Bucharest, Romania

**Keywords:** Biofilm formation, Staphylococcus aureus, Pseudomonas aeruginosa, quorum sensing, biofilm prevention.

## Abstract

Antibiotic resistance is a relevant topic nowadays, representing one of the main causes of infection-related mortality and morbidity at a global level. This phenomenon is worrisome and represents an area of interest for both clinical practice and fundamental research. One important mechanism whereby bacteria acquire resistance to antibiotics and evade the immune system is by forming biofilms. It is estimated that ~80% of the bacteria producing chronic infections can form biofilms. During the process of biofilm formation microorganisms have the ability to communicate with each other through quorum sensing. Quorum sensing regulates the metabolic activity of planktonic cells, and it can induce microbial biofilm formation and increased virulence. In this review we describe the biofilm formation process, quorum sensing, quorum quenching, several key infectious bacteria producing biofilm, methods of prevention and their challenges and limitations. Although progress has been made in the prevention and treatment of biofilm-driven infections, new strategies are required and have to be further developed.

## **SUMMARY**

**1. Introduction**

**2. Biofilm-producing bacteria**

*2.1 Staphylococcus aureus*

*2.2 Pseudomonas aeruginosa*

*2.3 Other bacteria*

**3. Biofilm development, quorum sensing and quorum quenching**

**4. Prevention of biofilm formation**

**5. Challenges and Limitations**

**6. Conclusion**

## **1. Introduction**

Bacterial biofilm is produced by ~80% of bacteria responsible for chronic infections and it is an important virulence mechanism, inducing resistance to antimicrobials and evasion from the host’s immune system^1^. The bacteria producing biofilm comprise a diverse group of organisms, including both Gram-negative and Gram-positive bacteria, aerobic and anaerobic, motile and non-motile, just to name a few.

Biofilm has a remarkable complexity and three-dimensional organization^2^ and forms when biofilm-producing bacteria in an aqueous environment adhere to solid surfaces and produce a network of extracellular polymeric substances (EPS), adopting a “multicellular lifestyle”^3^. These substances include but are not limited to: proteins, polysaccharides, lipids, DNA and form a protective matrix around bacteria, supporting their integrity and survival^4^. The microorganisms occupy about 10-30% of the biofilm volume. Approximately 97% of the biofilm is water, which is responsible for the flow of nutrients required for bacterial survival within the biofilmsl^5,6^. Some types of microorganisms first form aggregates of planktonic/free cells in an aqueous environment, as a first step inbiofilm formation^7^.

Biofilm is a useful adaptation of micro-organisms, enabling them to survive in certain environments^4^. Generally, microorganisms inside the biofilm are more difficult to eradicate than when present as single cells. This resilience is primarily due to the tolerance mediated by the biofilm-related extracellular network, metabolic dormancy and other potential mechanisms. Genetic factors do not play a major role in this type of resistance, although the close proximity of the cells may also facilitate the transfer of resistance genes^8^.

Both Gram-positive bacteria, such as *Staphylococcus aureus* and Gram-negative bacteria, such as* Pseudomonas aeruginosa* can be very difficult to eradicate when forming biofilms. Biofilm formation has significant implications and it is a serious problem in a few different fields, including healthcare/clinical care and food industry. In the hospital setting, there are specific bacteria, including *Staphylococcus epidermidis*, *Pseudomonas aeruginosa* and many others which colonize tissue from patients with chronic diseases, implants and/or catheters^4^. Most device-associated infections are due to microbial biofilm formation. In the food industry, the biofilm and the biofilm-producing bacteria can alter the food quality and compromise food safety. The biofilm can be found inside food recipients such as vats, mixing tanks or utensils used in food preparation^9^.

Current biofilm control strategies employed in both hospitals and food industry (e.g., cleaning, disinfection, surface preconditioning) are efficient to some extent. However, they are still far from the desired effect and control^4^, and biofilm-driven infections commonly recur. New strategies for targeting biofilms are thus required. One such strategy is the targeting of the quorum sensing system, which disrupts cell-to-cell communication, conjugation, nutrient acquisition and even motility and production of certain metabolites^4^.

## **2. Biofilm-producing bacteria and infections**

Based on the National Institute of Health (NIH)’s statistics, biofilm formation is present in about 65% of all bacterial infections and approximately 80% of all chronic infections (**Table 1**).

**Table 1:** Examples of bacterial species involved in biofilm formation and their biological effects

Bacterial strain	Gram stain	Types of infections	Reference
Staphylococcus aureus	Gram-positive	Chronic biofilm infections, right valve endocarditis, chronic wound infection, lung infections in patients with cystic fibrosis	^10-12^
Staphylococcus epidermidis	Gram-positive	Endocarditis, catheter-related infection, joint prosthesis infection	^13-15^
Streptococcus pneumoniae	Gram-positive	Lung infections, bacterial meningitis, acute or chronic otitis media	^16^
Listeria monocytogenes	Gram-positive	Co-culture interactions with Pseudomonas, Vibrio strains, listeriosis, contamination of food products	^17,18^
Burkholderia cepacia	Gram-negative	Opportunistic infections in patients with blood cancer	^19^
Escherichia coli	Gram-negative	Hemolytic uremic syndrome, acute diarrheic syndrome, urinary tract infections	^20^
Klebsiella pneumoniae	Gram-negative	Bacteremia, liver abscess, urinary tract infections	^21^
Pseudomonas putida	Gram-negative	Urinary tract infection	^22,23^
Pseudomonas aeruginosa	Gram-negative	Osteomyelitis, ventilator-associated pneumonia, lung infections in patients with cystic fibrosis, opportunistic infections in neutropenic patients, nosocomial infections	^10^
Pseudomonas fluorescens	Gram-negative	Bioremediation, biocontrol- Pythium, Fusarium, antimicrobial properties – production of mupirocin	^17,24-26^
Rhizobium leguminosarum	Gram-negative	Biocontrol properties – Pythium	^27^
Lactobacillus plantarum	Gram-positive	Prevention of Salmonella infection	^22,28^
Lactococcus lactis	Gram-positive	Antimicrobial properties in the human gastro-intestinal tract	^29^

Evaluation of the device-related infections resulted in several estimates, including 40% for ventricular-assist devices, 2% for joint prostheses, 4% for mechanical heart valves and 6% for ventricular shunts. Moreover, bacterial colonization of the indwelling devices was associated with infections in 4% of the cases when pacemakers and defibrillators were utilized, but also in 2% of breast implant cases^30-32^.

Infective valve endocarditis is an infection of the heart which usually occurs as the result of the adherence of bacteria to the endothelium. The most common germs involved in infective endocarditis are**staphylococci and streptococci, members of the HACEK group,**Gram-negative bacteria but fungal strains have also been described^33^. Seeding of the endothelium generally occurs from colonization or infection of different tracts, for example the genitourinary and gastrointestinal tract^34^, or through direct crossing of the skin barrier either due to wounds or through injecting drug use.

Other types of biofilm-driven infections include chronic wounds, diabetic foot infections, or pulmonary infections in patients with cystic fibrosis, to name only a few.

### *2.1 Staphylococcus aureus*

*Staphylococcus aureus* is a Gram-positive coccus that causes infections in certain conditions and is also part of the normal flora of the human body, such as the skin or the nasal mucosa.**The Centers for Disease Control and Prevention report that methicillin-resistant *Staphylococcus aureu*s (MRSA) is in top two typical hospital-acquired infections in the USA^35^.

Recently, a staphylococcal aggressiveness score has been defined, based on the presence of three main characteristics: tetrad formation, aggregative adherence and resistance to methicillin. While higher scores are associated with fulminant infection, lower scores are seen in biofilm-drive and relapse-prone infections^36^.

### *2.2 Pseudomonas aeruginosa*

*Pseudomonas aeruginosa* is a Gram-negative rod, facultatively anaerobe, present in a wide range of environments, including a part of the normal human flora, such as the gut flora. It commonly presents resistance towards multiple antibiotics, such as cephalosporins and, potentially, carbapenems, leading to extremely drug-resistant (XDR) infections**, **where the association of colistin to the antimicrobial regimen generally including a carbapenem such as meropenem may lead to synergic activity^37^. However, colistin has been associated with neurotoxicity and nephrotoxicity.

*Pseudomonas aeruginosa*’s potential of forming biofilm on medical device surfaces makes it a frequent agent of ventilator-associated pneumonia, or of other device-related infections, for example catheter-associated urinary tract infections^38^.

### *2.3 Other bacteria*

Many types of bacteria can produce biofilms, and some can also be involved in hospital-acquired infections. Examples include *Staphylococcus aureus*, *Pseudomonas aeruginosa*, *Escherichia coli*, *Klebsiella pneumoniae*, *Acinetobacter baumannii*, *Clostridioides difficile* and* Enterococcus* spp^38^. Other examples of biofilm forming bacteria are presented in **Table 1**.

## **3. Biofilm development, quorum sensing and quorum quenching**

**Figure 1:**
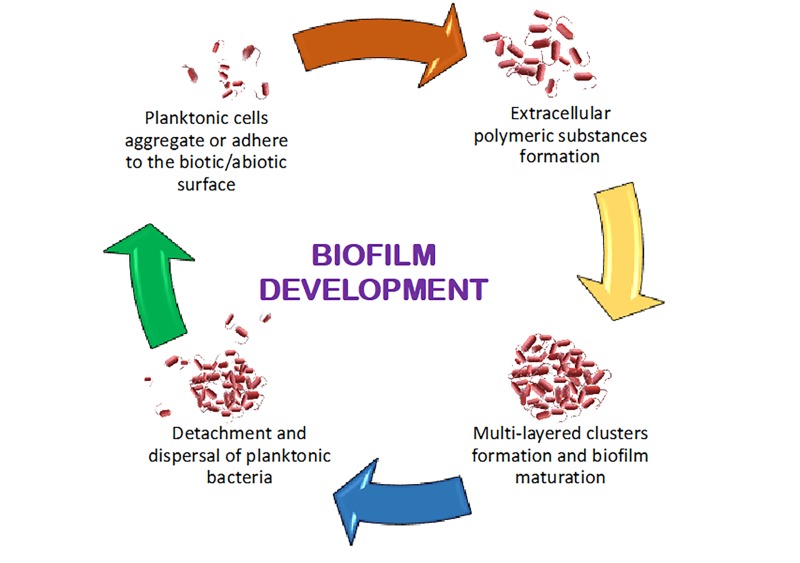
Biofilm Development

Formation of the biofilm comprises several steps, namely, the attachment, cell-to-cell adhesion, expansion maturation and dispersal (**Figure 1**)^4^. Bacterial multiplication leads to the development of microcolonies, which become encapsulated in a layer of hydrogel, that functions as a boundary between the microbial community and the external environment. **Table 2** indicates the main characteristics of the biofilm formation phases. Within the bacterial community, cells communicate with each other through quorum sensing (QS) systems, communication based on chemical signal. The role of communication is to modulate cellular functions, population density-based pathogenesis, nutrient acquisition, transfer of genetic material between the cells, motility and synthesis of secondary metabolites. The biofilm matures in parallel with the accumulation of extracellular polymeric substances. The final step involves the detachment of bacterial strains from the microcolonies, potentially leading to the formation of a new biofilm colony in a distinct location^38^.

**Table 2:** Particularities of biofilm formation phases

Phase	Characteristics
Adhesion of planktonic cells	Biofilms generally start by the adhesion of microbial cells to a biotic or abiotic surface. Biotic surfaces may include endothelial lesions, necrotic tissues, mucosae, etc., while abiotic surfaces may include indwelling devices: vascular catheters, urinary catheters; prostheses, surfaces from the clinical environment^39^. This surface adhesion, or primary attachment, can be active or passive depending on microbial factors such as motility, or expression of adhesins. Planktonic strains can move to a specific site and either adhere to an existing lesion/surface, or directly induce tissue necrosis, thereby favoring their subsequent adhesion. Cellular physiology changes, affecting surface membrane proteins. Removal of irreversibly attached cells is difficult as it requires use of specific enzymes, surfactants, sanitizers. Microbial adhesion is also influenced by the physicochemical properties of the biotic or abiotic surface. Bacteria behave as hydrophobic particles presenting negative charge, but this varies with growth phase^40^. While biofilms have classically been defined as surface-associated microbial cells, a revised definition of biofilms states that the essential characteristic of biofilms is actually inter-bacterial aggregation, which can also be independent of surface adhesion^41^.
Formation of an extracellular polymeric substance (EPS) matrix	Genes responsible for adhesion and matrix assembly are activated when stimulated by factors including population density and nutrient limitation. The EPS matrix is composed of a mixture of biopolymers. The matrix produced in broth culture is not similar to the one produced when strains are attached to a surface, and biofilms also differ between in vivo and in vitro conditions. EPS can also be produced by planktonic cells resulting in enhanced attachment^42^.
Accumulation of multi-layered clusters of microbial cells	Microcolony development is the result of simultaneous bacterial aggregation and growth. The tulip biofilm arrangement was established as a discrete model, using confocal laser microscopy^43^. This discrete model indicates that cells in the outer biofilm layers display active metabolism, while cells deeper inside the biofilm downregulate their metabolism and enter a dormant, persistent state.
Biofilm maturation	During biofilm maturation, canals are created in the biofilm structure. These will allow gradient-based passage of nutrients and signaling molecules, favoring organized agglomeration and differentiation of cells based on their metabolic state^42^.
Detachment and dispersal of planktonic bacteria	Following maturation, biofilms become thicker, developing an anaerobic environment on the interior, while external layers may begin separating. Detachment and dispersal can also occur when there is a nutritional imbalance. For instance, low carbon availability increases EPS synthesis^44^. Detached cells or clusters of cells can travel as septic emboli, and may colonize new sites, then generating infection with potentially new biofilm formation.

Organic extracellular molecules are produced by the microbial strains within the biofilms and released as both soluble (soluble microbial products (SMPs)) and insoluble materials (organic extracellular polymeric substances (EPS)) in the extracellular media^45,46^.**These substances originate from the substrate metabolism, being microbial byproducts and waste, but also cellular residual content from damaged cells. Insoluble materials or EPS are polysaccharides, extracellular DNA (eDNA) and/or proteins secreted by strains during the establishment and life of biofilms. Yet, most often the difference between the secreted molecules and those composing the microbial biofilm in not obvious^6,47,48^.

As biofilms have captured the attention of the scientific community, this extensive research field demands new data management and deeper analysis methodologies. The Minimum Information About a Biofilm Experiment (MIABiE) initiative (http://www.miabie.org), brings together an international group of experts, working on the development of guidelines to document bacterial biofilms investigations, as well as the standardization of the current nomenclature, the development and improvement of oriented computational resources and toolsfor deep-understanding, as well as for targeted biofilm research^49,50^.

### *Quorum Sensing*

Gram-positive bacteria use oligopeptides as signaling molecules to form biofilms, using QS for intraspecies communication. The QS system is a paramount target for the treatment of biofilm associated infections^51^. There are at least three main types of QS systems to be distinguished: the acyl homoserine lactone QS system (AHL) in Gram-negative bacteria, the autoinducing peptide (AIP) QS system in Gram-positive bacteria and the autoinducer-2 (AI-2) system in both Gram-negative and -positive bacteria^52^.

Homoserine lactones are a class of important cellular signaling molecules involved in QS and acyl homoserine lactone-dependent QS system is used primarily by Gram-negative bacteria. The AHL molecules have in common the homoserine lactone ring, although they vary in length and substitutes. Remarkably, AHLs are synthesized by a specific cognate AHL synthetase. Interestingly, an increased concentration of AHL was correlated to a significant bacterial growth^53^.

AIPs are signal molecules secreted by membrane transporters and synthesized by Gram-positive bacteria. As the environmental concentration of AIPs increases, these AIPs bind to the histidine kinase sensor, which phosphorylates, and as a consequence alters target gene expression. In *Staphylococcus aureus* quorum sensing signals are stringently regulated by the accessory gene regulator or *agr* which is associated with AIPs secretion. These genes are responsible for the production of numerous toxins and degradable exoenzymes.

As part of their cooperation and communication, microorganisms have the ability to sense and translate the signals from distinct strains in AI-2 or autoinducer-2 interspecific signals, catalyzed by LuxS synthase. Moreover, LuxS is involved in the activation of the methylation cycle, being demonstrated to control the expressions of hundreds of genes associated with the microbial processes of surface adhesion, detachment, and toxin production^54^.

## **4. Prevention of biofilm formation**

Both in healthcare and the food industry, the main strategy to tackle bacterial biofilms is to prevent their development. In order to do this, there are several ways (e.g., cleaning, sterilization) of preventing bacteria from reaching or proliferating in critical locations. In many cases, especially in food processing, sterility of the environment is not entirely possible and is not cost-effective. Measured taken involve thermal, chemical or mechanical strategies for bacterial biofilm prevention.

However, even with the best existing prevention measures adopted, biofilm may form and biofilm-producing bacteria could potentially be a problem. Thus, efficient diagnosis and treatment of biofilm-related infections is important in clinical settings. Several recommendations and guidelines exist and we recommend the summary presented Kamaruzzaman NF et al^38^. For example, the European Society for Clinical Microbiology and Infectious Diseases provides guidelines for the diagnosis of these infections, which implies both laboratory and clinical diagnosis methods^55^.

## **5. Challenges and Limitations**

Despite important advances in our current knowledge on biofilm-producing bacteria and the organization and function of the biofilm itself, significant research remains to be performed. This is in part due to the prior focus on the investigation of planktonic bacteria, not on the biofilm formation^4^. Together with this adaptation of the research focus, new methods (imagistic or molecular) are now available and under development for the investigation of biofilms and their components. Moreover, *in silico* methods may be a good solution in solving medical or technological challenges related to biofilm formation, prevention or treatment. Such methods comprise the biofilm consortia metabolic models, designed for the prediction of biofilm formation stages based on the components and their concentration or the molecular docking software for designing and predicting the efficiency of new drugs^56,57^.

New prevention and treatment strategies have to be further developed. **Table 3** summarizes the main mechanisms of resistance to antibiotics. The ability of biofilm-forming bacteria to adapt to the human environment is also related to immune system evasion. Bacteria from the biofilm can avoid recognition by immune system and avoid phagocytosis. Biofilm can obscure recognition of bacterial products such as lipopolysaccharides, lipoproteins and nucleic acids. Neutrophils migrate towards the biofilm produced by bacteria, such as *S*. *aureus* and *P. aeruginosa*. However, decreased phagocytosis and bacterial cell killing is observed upon this migration. Interestingly, the glycolipids implicated in quorum sensing by *P. aeruginosa* can induce necrosis of neutrophils and disruption of this process by using quorum sensing inhibitors promotes phagocytosis. Thus, new biofilm targeting treatments addressing evasion from the immune system can be developed^38^.

An important limitation in biofilm prevention, treatment and investigation is its physiological heterogeneity. Although individual cells can be isolated and investigated from the biofilm, the spatial relation, structure and properties are not preserved^50^. Thus, it is imperative to use techniques which preserve the spatial relationships between cells. The characteristics and physiology of bacterial cells from the biofilm can significantly differ based on their localization within the biofilm. This is not only important in investigation of the biofilm, e.g., -omic profiling resulting in average results for a diverse biofilm population but can also contribute to biofilm resistance to preventive and treatment measures^50^.

However, although many of the biofilms are regarded as harmful to humans, biofilms can also play a useful role, contributing for example to the genetic and natural diversity through cell to cell interactions within the biofilm, of protecting different organisms (e.g., marine algae) against pathogens^58^.

Control of the formation of biofilms that have negative effects on human health remains a challenge, with few treatment options clinically available.

**Table 3:** Mechanism of biofilm-mediated antimicrobial resistance

Resistance mechanism	Characteristics	Refs.
Glycocalyx	The capsule can be found in Gram-positive as well as in Gram-negative bacteria, being an integral part of the biofilm. The contribution to the maturation step is possible due to electrostatic and hydrogen bonds established between the matrix and the abiotic surface. Its composition in glycoprotein and polysaccharides varies with biofilm development, supporting pathogens to survive in adverse conditions. The antibiotic bacterial resistance and different units of antimicrobials are supported by the glycocalyx. The external layer acquires antimicrobial compounds, serving as well as adherent for exoenzymes and protecting against antibacterial activity by providing a substrate for biocides degradation.	^6,59^
Enzyme-mediated resistance	Enzymatic reduction of ionic particles mediates the transformation of toxic into nontoxic molecules. The existence of heavy metals, such as cadmium, nickel, silver, zinc, copper, cobalt, induces a large diversity of resistant phenotypes.	^60^
Metabolism and growth rate heterogeneity	Bacterial metabolic activity and growth rate are influenced by the heterogeneities in nutrients and the variable oxygen concentration within biofilms, having strong influence on the quantity of both metabolic substrates and products, especially at the peripheral area, where microbial proliferation is supported. Limited metabolic activity inside the biofilm results in slowly growing strains inside the matrix. The changes in cell growth cycle affect the enzymatic process inside biofilms, influencing both metabolic and growth rate variations. Moreover, microbial communities increase the level of antibiotic resistance by expressing certain genes under anaerobic conditions.	^61,62^
Cellular persistence	Persistent strains are responsible for the infections’ chronicity as they become tolerant to antibacterial agents. Biofilms contain persistent cells, which elicit multidrug tolerance. The glycocalyx improves the ability to protect the immune system, as they re-induce growth of bacterial biofilm and compete for the antibiotic targets and for multidrug resistance (MDR) protein synthesis.	^63-65^
Metabolic state	Nutrients’ limited availability affects the composition and modifies the prokaryotic envelope. After being exposed to inhibitory concentration of bactericidal agents, the resistant cell population shows phenotypic adaptation. Treating biofilms with antimicrobial agents conducts to loss of their respiratory activity.	^66,67^
Genetic profile	The multiple antibiotic resistance also known as mar operons are general regulators involved in control of various genes' expression in E. coli, supporting the MDR phenotype. Stress response cells show increased resistance to a damaging factor within hours of exposure. Diverse regulatory genes, for instance oxyR and soxR, were demonstrated to determine intracellular redox potential and activation of stress response when bacterial strains are exposed to molecular oxidizers.	^68,69^
Quorum sensing (QS)	QS influences the heterogeneous structure, as in convenient nutrient supply and suitable environment, the phenotype is essential in the cell migration process. QS deficiency was associated with thinner microbial biofilm development and, as a consequence, lower EPS production.	^70,71^
Stress response	The stress response acts as a preventive factor for cell damage more than repair. Starvation, decreased or increased temperature, high osmolality and low pH are seen as causes of stress induction. Altered gene expression due to the stress response in immobilized strains result in increased resistance to biocides.	^72-74^
External membrane structure	While most antibacterial agents must penetrate bacterial cells to target a specific site, modification of cellular membrane may control antibiotic resistance. The lipopolysaccharide layer prevents hydrophilic antimicrobials from entering through the outer membrane while the external membrane proteins reject hydrophobic molecules.	^75,76^
Efflux systems	Efflux pumps facilitate bacterial survival under extreme environmental conditions by exerting both intrinsic and acquired resistance to different antimicrobials, from the same or different families. Consequently, the overproduction of efflux pumps determines multidrug resistance, when combined with similar resistance mechanisms, for example antibiotic inactivation or target adjustment. The efflux pumps are seen as a major player in the MDR of Gram-negative bacteria because of the deep understanding of efflux pumps mechanisms which could provide drug discovery platforms in targeted bacterial pathogens.	^77-81^

## **6. Conclusion**

Biofilms enable bacteria to survive in specific environments, confer resistance or tolerance to treatment and the capacity to evade the host immune system. They represent a challenge in prevention and treatment of infections. Biofilms also confer to bacteria the property to resist standard cleaning procedures in the food industry. Thus, it is important to better understand how it can be prevented and managed and to develop effective targeted therapies.

Investigation of biofilm’s complex 3D structure, function, development, maturation and all characteristics of involved cells (proteomic, genomic data) is required for a better understanding of these processes. Attacking the biofilm should take into consideration not only targeting the bacteria inside the biofilm but also its extracellular components. Drug design, delivery and *in silico* methods can be used to predict or measure the efficacy of anti-microbial drugs when biofilm is present.

New anti-biofilm strategies are required and have to be further developed. These new treatments have to present high specificity, low toxicity on normal eukaryotic cells and host microbiota and be efficient in treating infection caused by the biofilm-causing organisms.**

## **KEY POINTS**

**◊** **Approximately 80% of the bacteria producing chronic infections can form biofilms, inducing resistance to antibiotics and immune system evasion**

**◊** **Quorum sensing regulates the metabolic activity of planktonic cells, and it can induce microbial biofilm formation and increased virulence**

**◊** **New strategies are required in order to target the biofilm and biofilm-producing bacteria**
